# Ailanthone induces apoptosis in U-2OS cells through the endoplasmic reticulum stress

**DOI:** 10.3389/fimmu.2025.1633643

**Published:** 2025-10-02

**Authors:** Yue Zhang, Taiding Wu, Chang Li, Nianfang Luo, Jun Wang, Jingxian Li, Min Tian, Lei Liu, Ruiting Li, Jingyi Zhang

**Affiliations:** ^1^ Department of Cardiology, Hubei No. 3 People’s Hospital of Jianghan University, Wuhan, Hubei, China; ^2^ Health Science Center, Hubei Minzu University, Enshi, Hubei, China; ^3^ Department of Critical Care Medicine, Union Hospital, Tongji Medical College, Huazhong University of Science and Technology, Wuhan, Hubei, China

**Keywords:** Ailanthone, osteosarcoma, endoplasmic reticulum stress, unfolded protein response, apoptosis

## Abstract

**Background:**

Osteosarcoma (OS) is a malignant bone tumor that commonly occurs in children and adolescents, characterized by poor treatment outcomes and prognosis, highlighting the urgent need for alternative therapies. Endoplasmic reticulum stress (ERS) induces a series of cascade reactions known as the unfolded protein response (UPR), which is a crucial stress mechanism that cells utilize to cope with disrupted endoplasmic reticulum function and is widely involved in the apoptosis of tumor cells. Excessive UPR can lead to an overload of protein levels within cells, disrupting homeostasis and exhausting energy, ultimately inducing apoptosis in OS cells. Ailanthone (AIL), a natural compound derived from the root bark or stem bark of *Ailanthus altissima* (Mill.) Swingle, exhibits broad-spectrum anticancer activity across multiple tumor types. Its antitumor mechanism involves the modulation of endoplasmic reticulum stress (ERS)-associated proteins, including the upregulation of apoptotic markers (PERK, eIF2α, ATF4, CHOP) and pro-apoptotic factors (BAX, caspase-3, Bim), alongside the downregulation of the anti-apoptotic protein BCL-2. This study aims to investigate whether AIL induces apoptosis in OS cells via ERS.

**Materials and methods:**

The effects of AIL (0-1.0 µmol/L) on the proliferation and migration of U-2OS cultured for 24 h were evaluated using the cell counting kit-8 assay and scratch wound healing assays. The optimal concentration of 0.6 µmol/L was selected for subsequent experiments. Western blot analysis was performed to measure the protein levels of ERS-related factors at different time points (0–24 h) following AIL treatment. Finally, the apoptosis rate of U-2OS cells after 24 h of culture at the optimal concentration was assessed by flow cytometry.

**Results:**

AIL exhibited a dose-and time-dependent inhibitory effect on U-2OS cell growth, significantly reducing cell proliferation and migration rates while promoting apoptosis. After AIL treatment, the levels of ERS-related proteins and pro-apoptotic proteins increased, while anti-apoptotic protein level decreased.

**Conclusion:**

AIL inhibited the proliferation of human OS cells and induced apoptosis through the ERS pathway. It represented a potential therapeutic agent for OS treatment.

## 1 Introduction

Osteosarcoma (OS), one of the earliest identified tumors in human history, has been recognized for over 1.7 million years and is the most common primary malignant bone tumor (1). The majority of cases occur in children and adolescents aged 10–30 years, with the highest incidence during periods of rapid growth in puberty (2). Approximately 10-15% of newly diagnosed OS patients present with metastatic disease, and more than 90% of OS-related deaths are due to lung metastasis (3). The standard treatment for osteosarcoma includes targeted radiation therapy, systemic chemotherapy, and surgical resection (4). However, these treatment options have shown limited efficacy, highlighting the urgent need for new targeted drug therapies as alternatives.

The endoplasmic reticulum (ER) is the site of protein synthesis, processing, transport, secretion, modification, and lipid and carbohydrate metabolism, as well as a primary calcium storage location (5). Disruptions in protein folding or secretion can lead to the accumulation of misfolded or unfolded proteins in the ER, a phenomenon known as endoplasmic reticulum stress (ERS). The subsequent protective signaling cascade is termed the unfolded protein response (UPR) (6). The UPR is crucial for maintaining tumor cell growth, survival, differentiation, and protein homeostasis, responding to various external stimuli leading to ERS (7). UPR is primarily regulated by three ERS sensors: protein kinase R-like endoplasmic reticulum kinase (PERK), activating transcription factor 6 (ATF6), and inositol-requiring enzyme 1α (IRE1α), which typically associate with binding immunoglobulin protein (BiP, also known as GRP78) to remain inactive (8). Under ERS, misfolded proteins interact with BiP, causing the dissociation of PERK, eukaryotic translation initiation factor 2 subunit alpha (eIF2α), and activating transcription factor 4 (ATF4) from Bip. A critical pathway involves PERK activation, leading to eIF2α phosphorylation and ATF4 activation. In the tumor microenvironment, persistent and intense ERS can trigger the transcription of C/EBP homologous protein (CHOP), a hallmark protein for ERS-induced apoptosis. Elevated CHOP levels subsequently downregulate anti-apoptotic B cell lymphoma 2 (BCL-2) family members, while pro-apoptotic proteins such as caspase-3, BCL-2-associated X protein (BAX), and BCL-2 interacting mediator of cell death (Bim) are aberrantly expressed, driving apoptosis.

ERS and UPR play significant roles in various cellular processes, influencing cellular states such as proliferation, autophagy, apoptosis, and drug resistance, with ERS critically affecting tumor cell apoptosis (9). The PERK/eIF2α/ATF4/CHOP signaling pathway and its associated caspase cascade are common mechanisms for inducing apoptosis in human OS cells, making the study of ERS-induced UPR in OS a key focus and providing new theoretical insights for improving clinical outcomes.

Plant-derived natural products and compounds have been used in clinical treatment, and herbal remedies are also employed in cancer therapy. Ailanthone (AIL), a pentacyclic diterpenoid lactone extracted from the traditional herb Ailanthus, has demonstrated significant clinical effects against inflammation, malaria, allergies, tuberculosis, ulcers, amoebic diseases, along with anti-cancer properties in various tumors. AIL has been reported to induce apoptosis in tumor cells by targeting multiple molecular proteins and pathways. For instance, it inhibits cell proliferation and induces deoxyribonucleic ccid (DNA) damage in gastric cancer models (10, 11). AIL suppresses the growth and metastasis of non-small cell lung cancer via the up-frameshift protein 1 (UPF1)/growth arrest-specific 5 (GAS5)/unc-51 like autophagy activating kinase 1 (ULK1) signaling pathway, and it reduces colon cancer cell viability through the signal transducer and activator of transcription 3 (STAT3) pathway (12, 13). Additionally, AIL modulates ERS-mediated UPR by affecting the expression of the PERK protein, regulating downstream CHOP, and impacting various targets, including caspase and BCL-2 family proteins, transcription factors (eg. β-catenin), tumor suppressor genes (eg. p53) and signaling pathways involved in cancer cell apoptosis (14). The BCL-2 family regulates mitochondrial apoptosis and promotes caspase cascade activation, upregulating pro-apoptotic proteins like BAX, caspase-9/3/7, and p53 while downregulating anti-apoptotic BCL-2 (15).

The three signaling pathways mediated by the UPR can all trigger pro-apoptotic cascades, activating downstream apoptosis-related proteins of caspase, further indicating a significant association between AIL and UPR-mediated apoptosis in OS cells (16). AIL may promote OS cell apoptosis by regulating the apoptotic cascade signaling pathway induced by ERS. Therefore, we hypothesized that AIL effects the proliferation and apoptosis of human OS cells, and induces apoptosis in human OS cells by modulating ERS.

## 2 Materials and methods

### 2.1 Cell culture

The human OS cell line (U-2OS) was obtained from the National Collection of Authenticated Cell Cultures (cat no. SC-SP-5030, Shanghai, China). U-2OS cells were seeded in 96-well culture plates at a density of 800 cells per well and maintained in U-2OS-specific complete growth medium (Pricella Biotechnology, Wuhan, China) under standard culture conditions (37°C, 5%CO_2_). Cells were treated with varying concentrations of AIL was sourced from Shanghai Aladdin Biochemical Technology (cat no. 981-15-7, purity>98%, Beijing, China) (0, 0.2, 0.4, 0.6, 0.8, and 1.0 μM). Cell proliferation was monitored periodically via phase-contrast microscopy (cat no. BX41-PHD-P11, Olympus, Tokyo, Japan), and subculturing was performed every 48 h.

### 2.2 Cell counting kit-8 assay

Cell viability was evaluated using the CCK-8 assay (cat no. RM02823, ABclonal, Wuhan, China). U-2OS cells were seeded into 96-well plates at a density of 1×10³cells per well. After 24 h of incubation, cells were treated with AIL at varying concentrations (0, 0.2, 0.4, 0.6, 0.8 and 1.0 μM). Subsequently, 10 μL of CCK-8 reagent was added to each well, followed by incubation for 24 h in a humidified atmosphere containing 5% CO_2_. The optical density (OD) at 450 nm was measured using a full-wavelength microplate reader (cat no. A51119600CC, Thermo Fisher Scientific, Waltham, MA, USA). The experiment included six treatment groups, each with three technical replicates, and the entire procedure was repeated in 4–6 independent biological triplicates.

### 2.3 Wound healing assay

U-2OS cells were uniformly seeded in 6-well plates and cultured for 24 h until reaching 90% confluency. The culture medium was aspirated, and a standardized linear scratch wound was generated perpendicular to the plate bottom using a sterile 1000μL pipette tip. The dislodged cellular debris was gently removed by washing three times with phosphate-buffered saline (PBS). Subsequently, serum-free medium (cat no. CM-0236, Pricella Biotechnology, Wuhan, China) was added, followed by treatment with AIL at graded concentrations (0, 0.2, 0.4, 0.6, 0.8 and 1.0 μM) for 24 h. Wound closure dynamics were documented at 0 h and 24 h using a laser scanning confocal microscope (cat no. FV4000, Olympus, Tokyo, Japan) with pre-calibrated reference marker scratch widths.

### 2.4 Western blotting

Following treatment with the selected concentration of AIL (0.6 μM) at indicated time points (0, 3, 6, 9, 12 and 24 h), total cellular proteins were extracted using an ultrasonic homogenizer (cat no. FB50, Thermo Fisher Scientific, Waltham, MA, USA). Protein samples (20 μL per well) were loaded onto 10% SDS-polyacrylamide gels (SDS-PAGE). Electrophoresis was performed at 90 V for 30 min followed by 120 V for 60 min. Proteins were then transferred to polyvinylidene fluoride (PVDF) membranes using a semi-dry transfer system (cat no. 1658033, Bio-Rad Laboratories, Hercules, CA, USA) at 120 V for 60 min. Membranes were blocked with 5% non-fat milk in Tris-buffered saline containing 0.1% Tween 20 (TBST) for 1 h at room temperature. Primary antibodies against PERK (1:1000 dilution, cat no. 3190, Cell Signaling Technology, Danvers, MA, USA), eIF2α (1:2000 dilution, cat no. 3398, Cell Signaling Technology, Danvers, MA, USA), ATF4 (1:1500 dilution, cat no. Ab270980, Abcam, Cambridge, UK), CHOP (1:5000 dilution, cat no. AB11419, Abcam, Cambridge, UK), BCL-2 (1:2500 dilution, cat no. 12789-1-AP, Proteintech, Rosemont, USA), caspase-3 (1:3000 dilution, cat no. 25128-1-AP, Proteintech, Rosemont, USA), BAX (1:5000 dilution, cat no. Ab32503, Abcam, Cambridge, UK), Bim (1:1500dilution, cat no. 22037-1-AP, Proteintech, Rosemont, USA) and β-actin (1:3000 dilution, cat no. 20536-1-AP, Proteintech, Rosemont, USA) were applied and incubated overnight at 4°C. Membranes were subsequently incubated with fluorescently labeled secondary antibodies (anti-rabbit IgG, 1:10,000 dilution, cat no. 81115-1-RR, Proteintech, Rosemont, USA) for 2h at room temperature. Protein bands were visualized using an Odyssey Infrared Imaging System (cat no. LI-COR Odyssey CLX, Licor, NE, USA), and band intensity was quantified using ImageJ software (National Institutes of Health, Bethesda, MD, USA). All antibodies were diluted in TBST buffer, which was freshly prepared before each use. The antibody labeling was visualized with a Super Signal West Pico Chemiluminescent Substrate according to the manufacturer’s instructions (cat no. A38556, Thermo Fisher Scientific, Waltham, MA, USA).

### 2.5 Flow cytometry

U-2OS cells were uniformly seeded in 6-well plates and cultured for 24 h to reach 80% confluency. Cells were treated with 0.6 μM AIL for 0, 12, and 24 h. After treatment, cells were harvested and washed twice with ice-cold PBS; Cell pellets were resuspended in 500 μL of 1× binding buffer, followed by staining with 5 μL Annexin V-FITC (cat no. 6592, Cell Signaling Technology, Danvers, MA, USA) and 10 μL propidium iodide (PI) for 5 min at room temperature in the dark. Apoptosis was quantified using a flow cytometer (cat no. SH800SEP, Sony Biotechnology, Tokyo, Japan) with fluorescence detection channels set at 488 nm excitation/530 nm emission (FITC) and 535 nm excitation/615 nm emission (PI). Data analysis was performed using FlowJo software (FlowJo LLC, Ashland, OR, USA).

### 2.6 Statistical analysis

All data were analyzed using GraphPad Prism 8.0.2 (GraphPad Software Inc, La Jolla, CA, USA). Continuous variables are presented as mean ± standard deviation (SD). Comparisons among multiple groups were performed using one-way analysis of variance (ANOVA), and the standard t-test for comparisons between two groups. P < 0.05 indicates a statistically significant difference. *P < 0.05, **P < 0.01, ***P < 0.001. Each experiment was independently repeated 4–6 times.

## 3 Results

### 3.1 AIL inhibits U-2OS cell viability

**Figure 1:**
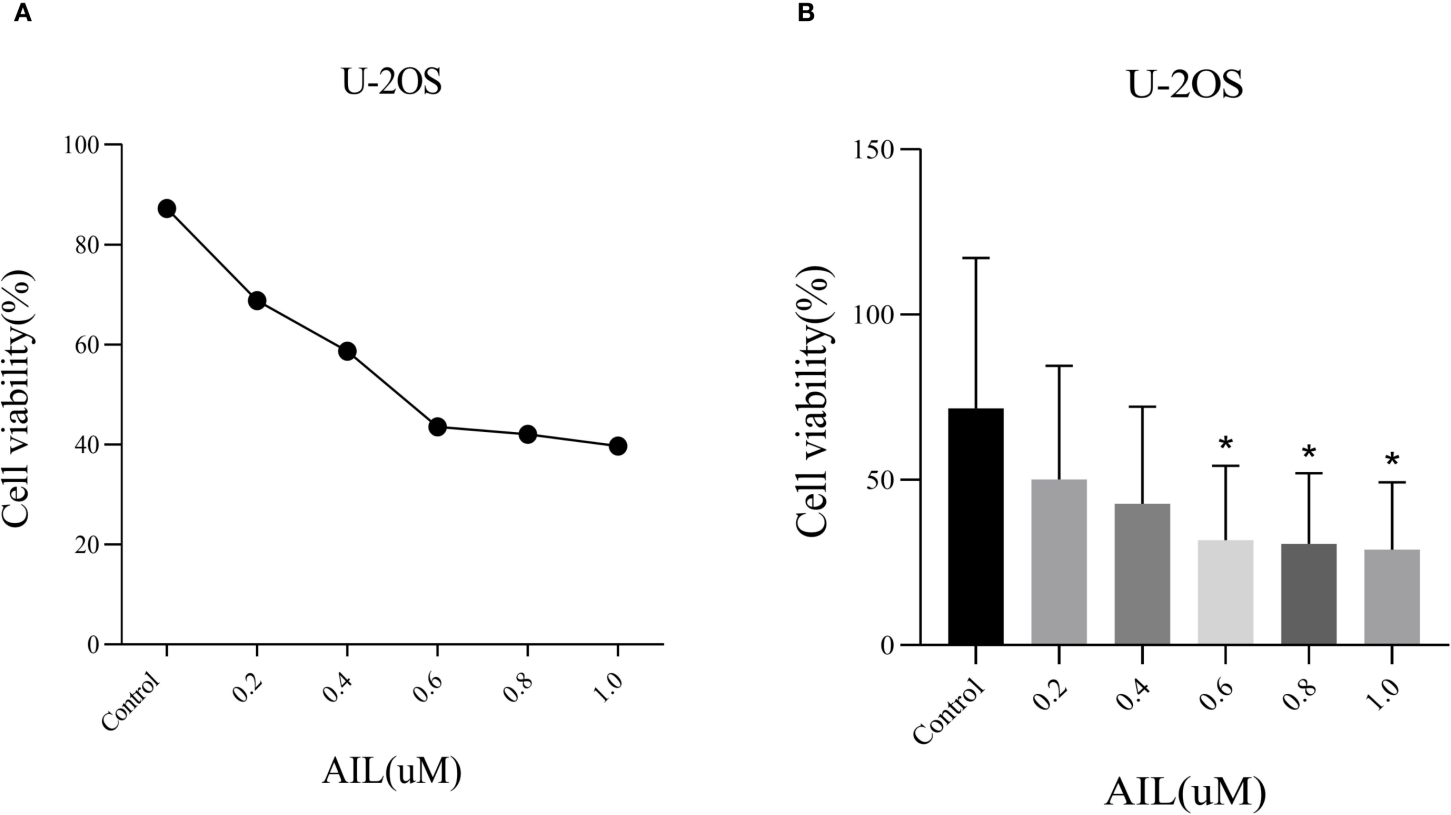
AIl inhibited U-2OS cells viability in a concentration-dependent manner. CCK-8 assay demonstrated that AIL (0 *vs* 0.2, 0.4, 0.6, 0.8 and 1.0 μM) treatment for 24 h reduced the viability of U-2OS cells **(A, B)** Data are presented as mean ± SD. n=4-6, *Compared with control group (0μM). 0.4 μM *vs* Control, p=0.0189; *p < 0.05. 0.6 μM *vs* Control, p=0.0173, *p < 0.05; 0.8 μM *vs* Control, p=0.0164, *p < 0.05.

CCK-8 assay results demonstrated that AIL significantly suppressed U-2OS cell proliferation in a dose-dependent manner (**Figure 1A**). Higher concentrations of AIL correlated with reduced cell viability. Treatment with 0.6 μM AIL induced an approximately 60% reduction in proliferation, with the inhibitory effect exhibiting time dependency (**Figure 1B**). These findings suggested that AIL suppressed U-2OS cell viability, and the magnitude of inhibition escalates with increasing drug concentrations.

### 3.2 AIL inhibits U-2OS cell migration

**Figure 2:**
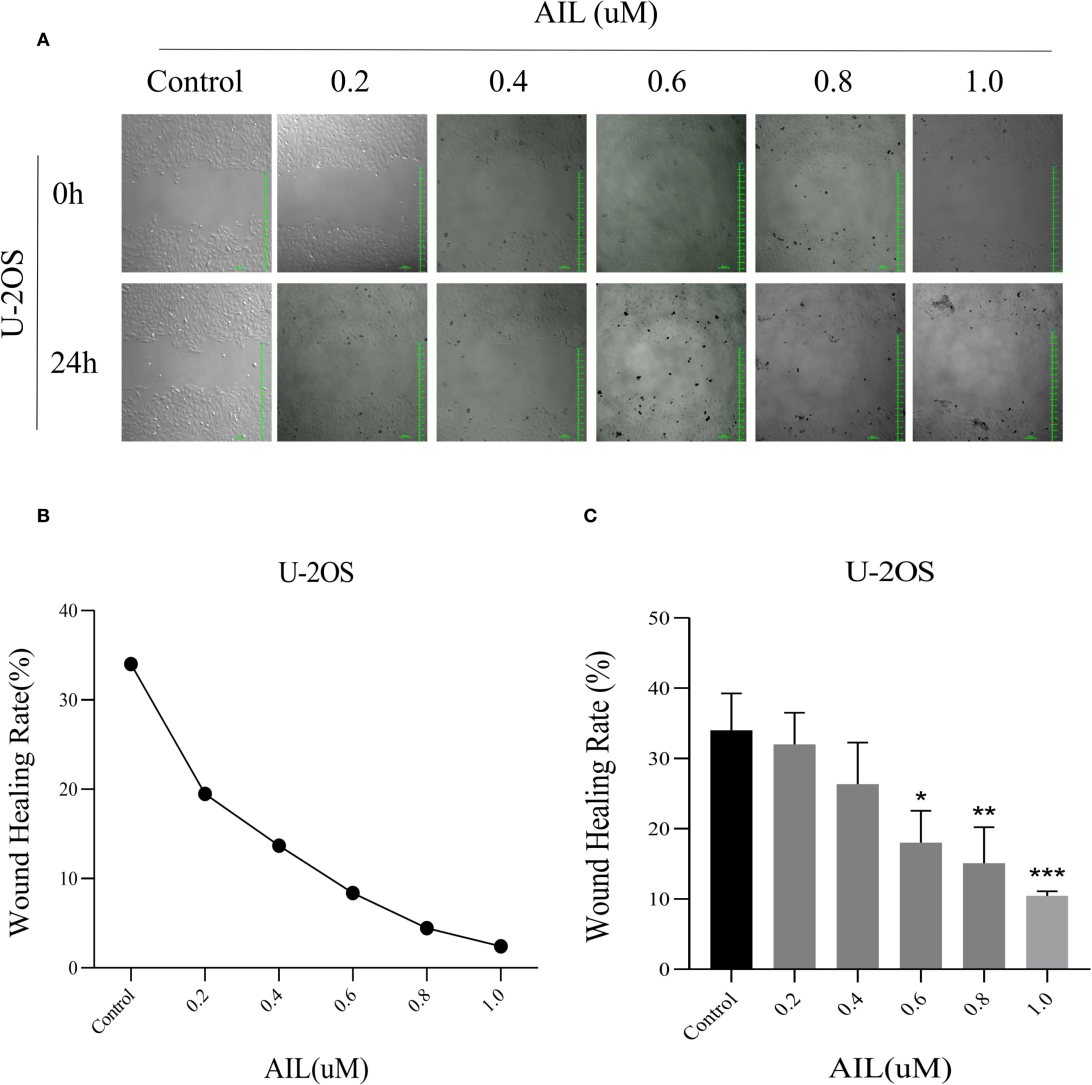
AIl inhibited U-2OS cells migration rate in a concentration-dependent manner. Wound healing assay demonstrated that AIL (0 vs 0.2, 0.4, 0.6, 0.8 and 1.0 μM) treatment for 24 h significantly inhibited the migration rate of U-2OS cells **(A–C)**. Data are presented as mean ± SD. n=4-6, *p < 0.05, **p < 0.01, ***p < 0.001. *Compared with control group (0 μM). 0.6 μM *vs* Control, p=0.0154; *p < 0.05. 0.8 μM *vs* Control, p=0.0044, **p < 0.01; 1.0 μM *vs* Control, p=0.0006, ***p < 0.001.

Wound healing assays revealed that AIL treatment (0, 0.2, 0.4, 0.6, 0.8 and 1.0 μM) for 24 h progressively reduced cell migration rates in a concentration-dependent manner (**Figure 2A**). Notably, at 0.6 μM of AIL, wound closure rates were markedly diminished (**Figures 2B, C**). These data indicated that AIL inhibited U-2OS cell migration, with efficacy proportional to drug concentration.

### 3.3 AIL upregulates ERS-related PERK/eIF2α/ATF4/CHOP protein expression

**Figure 3:**
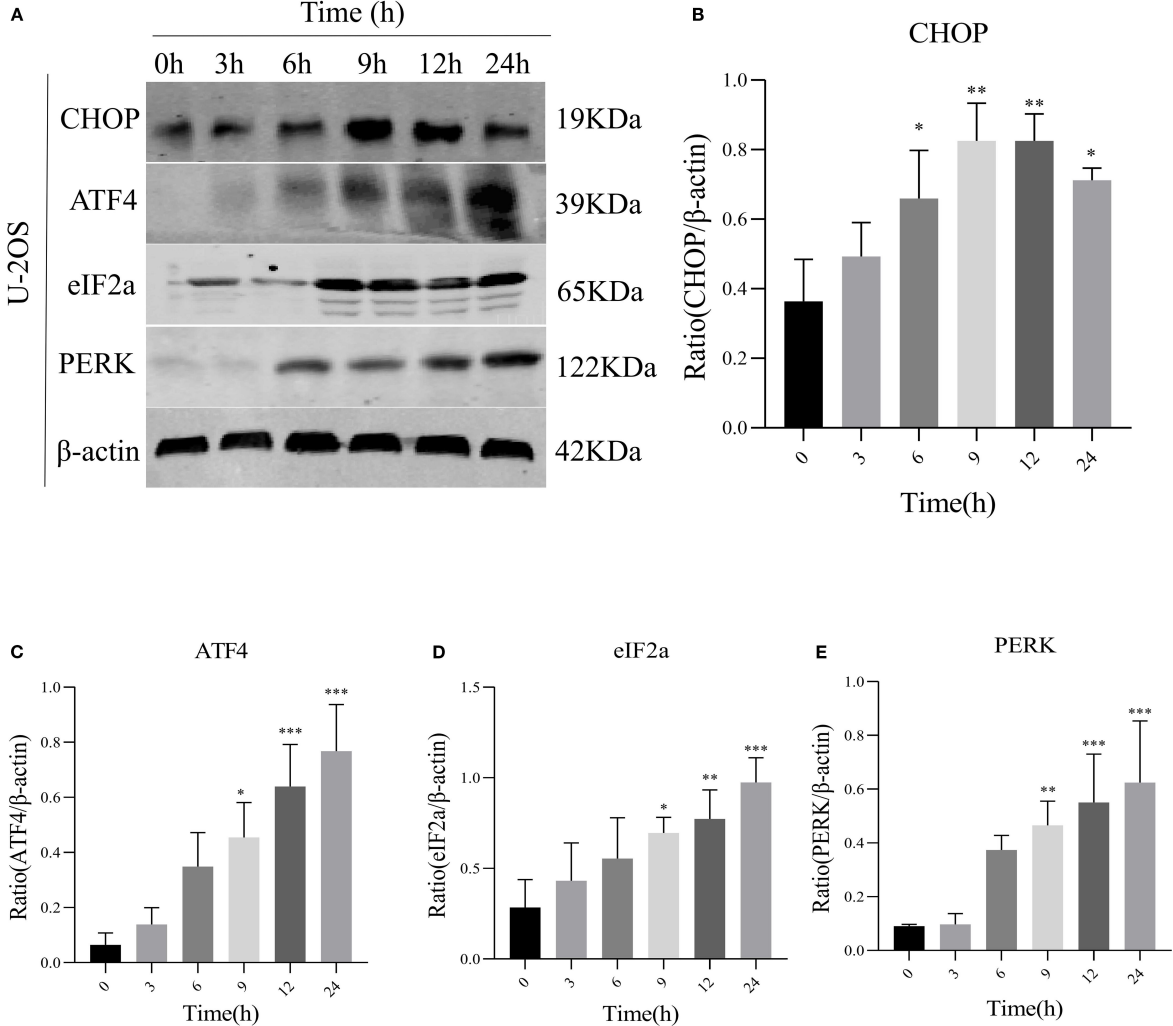
AIL promoted the expression of ERS-related proteins PERK, eIF2α, ATF4, and CHOP. WB assay analysis the protein expression levels in U-2OS cells after AIL (0.6 μM) treatment at different time points (0 h *vs* 3 h, 6 h, 9 h, 12 h and 24 h) **(A–E)**. Data are presented as mean ± SD. n=4-6, *p < 0.05, **p < 0.01, ***p < 0.001.*Compared with control group (0 μM). β-actin was used as an internal control.

We speculated a potential association between AIL and the ERS-mediated PERK/eIF2α/ATF4/CHOP pathway. Prior studies suggested AIL-induced tumor cell apoptosis may involve the UPR (17). To validate whether AIL activates the PERK/eIF2α/ATF4/CHOP axis, we quantified four key proteins. Following treatment with 0.6 μM AIL at varying time points (0, 3, 6, 9, 12 and 24 h), time-dependent upregulation of PERK, eIF2α, ATF4, and CHOP was observed (**Figures 3A–E**). These results suggested that AIL potentiated ERS by activating the canonical PERK/eIF2α/ATF4/CHOP UPR pathway.

### 3.4 AIL induces U-2OS cell apoptosis via UPR signaling

**Figure 4:**
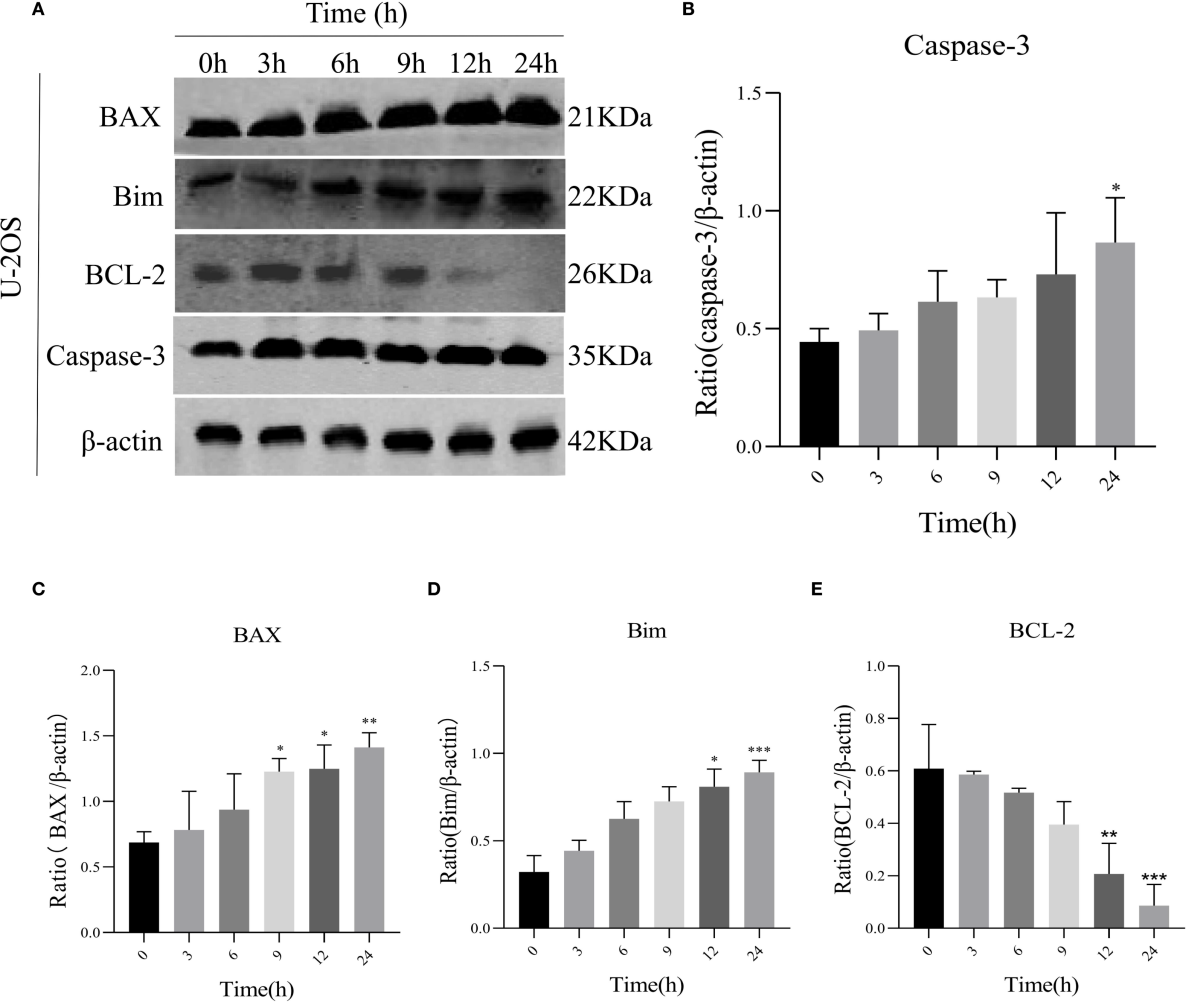
AIL promoted U-2OS cell apoptosis via ERS-related proteins. WB assay analysis the expression of apoptosis-related proteins BAX, caspase-3, Bim, BCL-2 in U-2OS cells after AIL (0.6 μM) treatment at different time points (0 h vs 3 h, 6 h, 9 h, 12 h and 24 h) **(A–E)**. Data are presented as mean ± SD. n=4-6, *p < 0.05, **p < 0.01, ***p < 0.001.*Compared with control group (0 μM). β-actin was used as an internal control.

**Figure 5:**
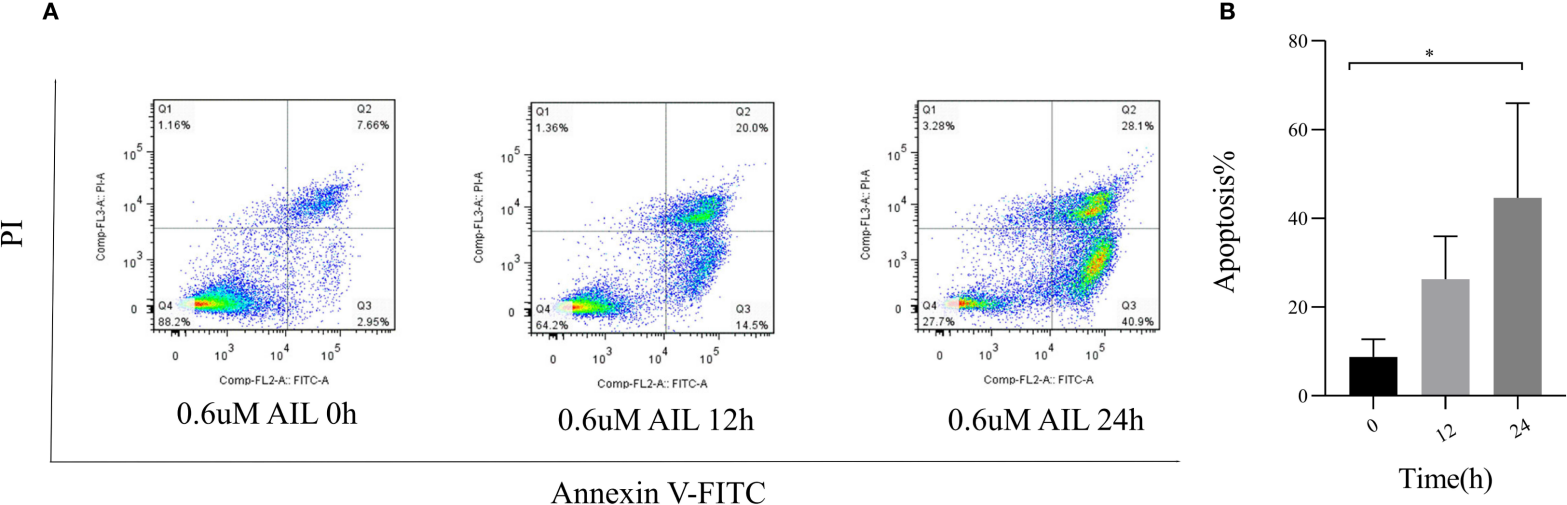
AIL promoted the apoptosis of U-2OS cells. Flow cytometry assay demonstrated the apoptosis rate of U-2OS cells after AIL (0.6 μM) treatment at different time points (0 h vs 3 h, 6 h, 9 h, 12 h and 24 h) **(A, B)**. Data are presented as mean ± SD. n=4-6, *p < 0.05. *Compared with control group (0 μM).

To assess AIL’s pro-apoptotic effects, we analyzed apoptosis-related proteins. WB analysis revealed that AIL upregulated pro-apoptotic proteins caspase-3, BAX, and Bim, while downregulating the anti-apoptotic protein BCL-2 (**Figures 4A–E**). Flow cytometric analysis further confirmed that AIL treatment for 24 h significantly increased apoptotic rates compared to control group (**Figures 5A, B**). Both concentration- and time-dependent apoptosis induction were observed, consistent with WB results. These data collectively supported the hypothesis that AIL triggered apoptosis in U-2OS cells through PERK/eIF2α/ATF4/CHOP UPR pathway.

## 4 Discussion

Our study demonstrated that AIL exerted dose- and time-dependent inhibitory effects on U-2OS cell proliferation and migration while promoting apoptosis. Mechanistically, AIL induces apoptosis through activation of the PERK/eIF2α/ATF4/CHOP signaling axis, a pivotal branch of the UPR, coupled with initiation of the caspase cascade. These findings strongly suggested that AIL triggered OS cell apoptosis via ERS-mediated pathways.

OS remains a leading cause of cancer-related mortality in adolescents and young adults, with frequent metastatic dissemination and poor prognosis (18). Although synthetic inhibitors used in targeted therapies have improved clinical outcomes, their utility is limited by drug resistance and systemic toxicity. Current multimodal chemotherapy regimens achieve 5-year survival rates of 60%–70% in non-metastatic cases (19). However, for patients with recurrent or metastatic disease, survival rates stagnate at approximately 20% over the past three decades (20). This therapeutic stagnation underscores the urgent need to develop alternative biotherapeutics with multi-target efficacy and reduced off-target toxicity.

The ER is a pivotal organelle in eukaryotic cells, monitoring intracellular proteostasis, lipid metabolism, and calcium homeostasis through ERS. Cellular apoptosis isregulated by multiple molecular pathways, including the ERS-mediated UPR (21). The UPR comprises three major transmembrane sensors: PERK, ATF6, and IRE1α.These sensors remain inactive through binding to the molecular chaperone BiP/GRP78 (22). During tumor-associated ERS, BiP preferentially binds misfolded/unfolded proteins, leading to dissociation and activation of IRE1α, PERK, and ATF (23). Activation of the canonical PERK/eIF2α/ATF4/CHOP pathway occurs as follows: Misfolded proteins trigger PERK, inducing its oligomerization and auto-phosphorylation. Subsequently, PERK phosphorylates eIF2α, a ubiquitous translation initiation factor. phosphorylatedeIF2α (p-eIF2α) attenuates global protein synthesis, alleviating proteotoxic stress to promote tumor cell survival. However, persistent ERS activatesATF4-mediated transcriptional upregulation of CHOP, which drives pro-apoptotic gene expression. Excessive protein accumulation and energy depletion under chronic ERS ultimately trigger tumor cell apoptosis. Emerging evidence highlights the UPR’s critical role in modulating autophagy, apoptosis, inflammation, and oxidative stress in malignancies (24). For instance, pharmacologic targeting of GRP78/BiP and CHOP induces apoptosis in glioblastoma (25). In pancreatic cancer, UPR activation dictates cellular fate, with UPR-mediated autophagy enhancing chemotherapeutic efficacy. Similarly, UPR signaling is pivotal in osteosarcoma pathophysiology (26). Studies demonstrate osteosarcoma cell apoptosis via PERK/p-eIF2α/CHOP/mTOR and IRE1/TRAF2/ASK1/JNK axes. Combinatorial therapy with CYT997, bortezomib, and doxorubicin induces oxidative stress-mediated apoptosis through PERK/eIF2α/CHOP signaling. Surfactin triggers ERS-associated inositol-requiring enzyme 1 (IRE1)/apoptosis signal-regulating kinase 1 (ASK1)/c-jun N-terminal kinase (JNK) activation and caspase-dependent apoptosis in human OS cells, demonstrating antitumor activity. These findings collectively validate ERS induction as a promising therapeutic target for OS.

AIL, a bioactive compound extracted from the traditional Chinese medicinal plant *Ailanthus altissima*, has been historically utilized in Chinese herbal medicine (27). Recent studies have increasingly focused on its antitumor potential, as evidenced by reference. AIL exerts anticancer effects by modulating apoptosis-related molecules upregulating pro-apoptotic factors and downregulating anti-apoptotic ones to inhibit proliferation and induce cancer cell death. Prior research demonstrates that AIL triggers autophagic and apoptotic cell death in human promyelocytic leukemia HL-60 cells, suppresses growth and metastasis in castration-resistant prostate cancer, targets DNA replication via RPA1 downregulation in non-small cell lung cancer, and inhibits gastric cancer progression through PERK-mediated apoptotic signaling (28–30). These findings collectively position AIL as a novel and promising antitumor agent.

Notably, several phytochemicals such as curcumin inducing ERS to inhibit breast cancer proliferation, palmitic acid activating CD36-dependent ERS to provoke ferroptosis in colon cancer, and titanium dioxide nanoparticles promoting ERS-driven apoptosis in hepatocellular carcinoma-highlight the therapeutic potential of ERS modulation (31, 32). Similarly, AIL induces tumor apoptosis via PERK-mediated caspase cascades, paralleling the mechanism by which ERS-activated UPR triggers OS cell death. To validate whether AIL’s anti-osteosarcoma effects involve the PERK/eIF2α/ATF4/CHOP pathway, we investigated ERS-mediated apoptotic mechanisms.

Our experimental results demonstrated that AIL dose-dependently inhibited U-2OS cell proliferation and migration, as shown by CCK-8 and wound healing assays. WB analysis revealed significant upregulation of ERS related proteins-PERK, eIF2α, ATF4, CHOP, indicating hyperactivation of ERS and subsequent UPR failure. Concurrently, pro-apoptotic proteins-BAX, caspase-3, Bim were upregulated, while the anti-apoptotic protein BCL-2 was downregulated. Flow cytometry confirmed increased apoptosis rates in AIL-treated cells, substantiating that AIL induces U-2OS apoptosis via ERS-triggered UPR.

While the PERK/eIF2α/ATF4/CHOP axis appears central to AIL’s pro-apoptotic effects, additional mechanisms may contribute and warrant further exploration. Critically, the absence of clinical trials confines AIL’s antitumor efficacy to preclinical models. To advance its therapeutic development, multicenter randomized controlled trials are imperative.

## 5 Conclusion

This study establishes AIL’s capacity to suppress OS cell proliferation and migration while inducing apoptosis through ERS/UPR pathways. These findings not only validate AIL’s potential as an anticancer agent but also expand the mechanistic understanding of ERS-targeted therapies for OS, providing a foundational framework for future translational research.

## Data Availability

The original contributions presented in the study are included in the article/supplementary material. Further inquiries can be directed to the corresponding authors.
